# Assessment of smoking cessation outcomes in patients with cardiovascular disease: A retrospective cohort study from Türkiye

**DOI:** 10.18332/tid/203867

**Published:** 2025-05-19

**Authors:** Yagmur Gokseven Arda, Guzin Zeren Ozturk

**Affiliations:** 1 Department of Family Medicine, Şişli Hamidiye Etfal Training and Research Hospital, University of Health Sciences, Istanbul, Türkiye

**Keywords:** cardiovascular disease, nicotine dependence, smoking cessation

## Abstract

**INTRODUCTION:**

Cardiovascular disease (CVD) is a major preventable cause of mortality, and smoking cessation significantly reduces the risk of recurrent cardiovascular events. However, many patients continue smoking despite their CVD diagnosis. This study aims to evaluate the impact of CVD and associated factors on smoking cessation success in patients who attempted to quit smoking at least one year ago.

**METHODS:**

This retrospective cohort study included patients who applied to the Smoking Cessation Clinic (SCC) between 1 May 2022 and 30 April 2023. A total of 539 eligible patients were analyzed. Data on demographics, CVD status, Fagerström test for nicotine dependence score, treatment modality (bupropion, nicotine replacement therapy, behavioral counseling), and smoking cessation outcomes were collected from medical records and confirmed via telephone interviews. Smoking cessation success was defined as complete abstinence after one year.

**RESULTS:**

Among the participants, 145 (26.9%) had a history of CVD. The overall self-reported smoking cessation success rate after one year was 16.7%, and 17.2% in the CVD group. Bupropion users had significantly higher treatment completion rates (p=0.015). Multivariable logistic regression analysis revealed that older age (AOR=1.03; 95% CI: 1.01–1.05), higher level of education (AOR=1.84; 95% CI: 1.03–3.26), lower nicotine dependence score (AOR=0.85; 95% CI: 0.77–0.94), and treatment completion (AOR=0.13, 95% CI: 0.07–0.23) were significantly associated with smoking cessation success in the total sample. Among patients with CVD, older age (AOR=1.06; 95% CI: 1.01–1.12) and treatment completion (AOR=0.15; 95% CI: 0.05–0.43) were also associated with higher cessation success. Patients with CVD were more likely to receive non-pharmacological interventions, and behavioral counseling alone showed the highest success rate (25.0%).

**CONCLUSIONS:**

Older age and treatment adherence were significantly associated with smoking cessation success, yet overall cessation rates remained low. A CVD diagnosis alone did not significantly enhance success, highlighting the need for tailored behavioral support and structured follow-up. Optimizing cessation programs with individualized interventions may improve outcomes, particularly in high-risk CVD patients.

## INTRODUCTION

The tobacco epidemic remains one of the most significant public health challenges worldwide. Tobacco use is responsible for over 8 million deaths annually, with approximately 80% of tobacco-related morbidity and mortality occurring in low- and middle-income countries, where the burden is highest. Türkiye ranks among the leading countries in tobacco consumption and is considered a key nation within the World Health Organization’s tobacco control initiatives^1^. Over the past decade, the daily smoking prevalence among individuals aged ≥15 years in Türkiye has increased to 28.3%^2^.

In 2011, Türkiye launched the *Smoking Cessation Treatment Support Program*, which provides free smoking cessation treatments through Smoking Cessation Clinics (SCCs) across all provinces^3^. Despite the implementation of tobacco control regulations and cessation support programs, the success rates of smoking cessation in Türkiye remain lower than those reported in other European countries^4^.

Cardiovascular disease (CVD) was defined as the presence of one or more physician-diagnosed conditions including coronary artery disease, myocardial infarction, arrhythmia, heart failure, peripheral artery disease, or valvular disease^5,6^.

Smoking is the most preventable cause of CVD and doubles the risk of nearly all types of CVD^7^. Even in patients who have already experienced a cardiovascular event, smoking cessation significantly reduces the risk of recurrent cardiovascular events and mortality while improving overall health parameters^8^. Studies suggest that individuals who quit smoking after their first cardiovascular event may live, on average, five years longer than those who continue smoking^9^. Moreover, tobacco-related diseases such as cancer and chronic obstructive pulmonary disease (COPD) further compound the disease burden in CVD patients, reducing life expectancy and increasing healthcare costs^9,10^.

Although a CVD diagnosis is a strong motivator for smoking cessation, many patients continue smoking even after a critical cardiovascular event. This suggests that the difficulty of quitting smoking is influenced by numerous factors, making the cessation process particularly complex^11^.

This study aims to retrospectively evaluate the impact of CVD and related factors on smoking cessation success in patients who attempted to quit smoking at least one year prior.

## METHODS

### Study design, setting, and participants

This retrospective cohort study was conducted in Türkiye and included patients who applied to the Smoking Cessation Clinic of Şişli Hamidiye Etfal Training and Research Hospital, University of Health Sciences, between 1 May 2022 and 30 April 2023. The study was carried out in 2024 after obtaining approval from the institutional ethics committee. Patients who met the inclusion criteria were enrolled in the study.

Inclusion criteria were defined as having complete medical records, being reachable by phone, and consenting to participate in the study. Exclusion criteria included patients with incomplete data, those who had passed away, those who declined participation, and those who could not be reached despite multiple call attempts.

### Data collection and variable definitions

**Figure 1:**
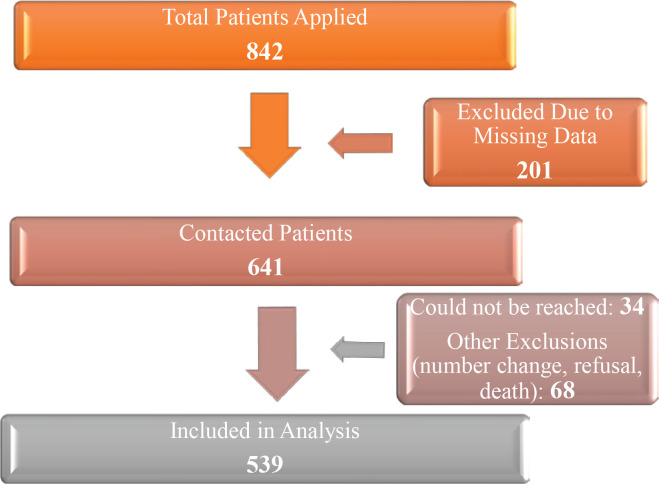
Patient flow diagram for the retrospective cohort study on smoking cessation outcomes among participants from a smoking cessation clinic in Türkiye, May 2022 – April 2023 (N=539)

A total of 842 patients applied to the SCC during the study period. Due to missing data, 201 patients were excluded from the analysis. The remaining 641 patients were contacted by telephone, but 34 patients could not be reached despite five repeated call attempts. Additionally, 68 patients were excluded due to phone number changes, refusal to participate, or death. Consequently, 539 patients who met the inclusion criteria were included in the final analysis (Figure 1).

Patient data were extracted from medical records, including age (years), gender (male, female), education level (high school or lower, university or higher), income status (minimum wage or lower, higher than minimum wage), presence of chronic diseases (present, absent), CVD diagnosis (myocardial infarction, coronary artery disease, atherosclerosis, heart failure, peripheral artery disease, arrhythmias, valvular heart diseases, cardiomyopathies), cigarette pack-years (cigarettes per day × years smoked/20), Fagerström test for nicotine dependence (FTND) scores (range: 0–10), and smoking cessation treatments received.

Treatment modalities included bupropion, nicotine patch, nicotine gum, combination therapies, and behavioral counseling. Behavioral counseling was provided to all pharmacologically treated patients. The non-pharmacological group included patients who received only behavioral counseling, while the combination therapy group included those who received multiple pharmacological treatments simultaneously. Patients in the combination therapy group were not included in monotherapy subgroups (bupropion, nicotine gum, patch).

All pharmacological treatments were prescribed in accordance with national smoking cessation guidelines^12^. In the nicotine gum group, patients received 2 mg or 4 mg formulations based on their level of nicotine dependence. However, exact dosage may have varied depending on physician assessment. In the patch group, standard 16-hour patches of 25 mg, 15 mg, or 10 mg were used. Behavioral counseling consisted of individualized face-to-face sessions supported by follow-up visits or calls.

Patients were contacted via telephone to verify their data, assess treatment adherence, and determine their smoking cessation status. Smoking cessation success was defined as complete abstinence from smoking for one year, based on self-reported data. Smoking cessation success was classified into two groups: successful (complete cessation) and unsuccessful (never quit or relapsed). Treatment completion was classified into two groups: completed treatment and discontinued treatment. Treatment adherence was defined as at least two months of bupropion use or three months of nicotine replacement therapy; for behavioral counseling, adherence was based on follow-up attendance documented in clinic records.

All patients were first compared in terms of smoking cessation success and treatment completion. Subsequently, they were categorized into two groups based on the presence or absence of CVD. Finally, factors influencing smoking cessation success were analyzed exclusively within the CVD group.

The Fagerström test for nicotine dependence test (FTND) is the most widely used tool for assessing nicotine dependence. The Turkish validity and reliability study of FTND was conducted by Uysal et al.^13^. The test consists of six questions, classifying nicotine dependence according to the scores: 0–2 very low, 3–4 low, 5 moderate, 6–7 high, and 8–10 very high.

### Statistical analyses

All statistical analyses were performed using SPSS version 25.0. Descriptive statistics are presented as frequency and percentage for categorical variables, and mean, standard deviation, and range for numerical variables. The Kolmogorov-Smirnov test was used to assess the normality of continuous variables. For normally distributed numerical variables, the Independent t-test was used for twogroup comparisons, while the Mann-Whitney U test was used for non-normally distributed numerical variables; the Independent t-test was used for two-group comparisons. For normally distributions, group comparisons for continuous were conducted using the one-way ANOVA test. The chi-squared test was used to compare categorical variables. Adjusted standardized residuals were used to interpret the contribution of specific cells to overall chi-squared significance. Multivariable logistic regression analysis was performed to identify factors associated with smoking cessation success. Multivariable logistic regression models were constructed using a manual stepwise approach. Variables with a p<0.05 in a univariate analysis and those considered clinically relevant (e.g. age, education level, treatment adherence) were included. Confounders were selected based on their theoretical and empirical relevance. The final model was selected based on Nagelkerke R^2^ and interpretability. A p<0.05 was considered statistically significant.

### Ethical approval

The study was designed as a retrospective cohort study, and ethical approval was obtained from the Clinical Research Ethics Committee of Şişli Hamidiye Etfal Training and Research Hospital, University of Health Sciences, on 28 May 2024 (Approval No: 4415).

## RESULTS

A total of 539 patients were included in the analysis. The mean age of the participants was 41.69 ± 12.40 years (range: 17–76). The mean cigarette pack-years was 26.92 ± 19.48 (range: 1–150), and the mean FTND score was 6.26 ± 2.51 (range: 0–10); 310 participants (57.5%) were male, and 351 (65.1%) had an education level of high school or lower; 241 patients (44.7%) had an income at or below the minimum wage, while 300 participants (55.7%) had no chronic diseases. A history of CVD was present in 145 patients (26.9%).

**Figure 2:**
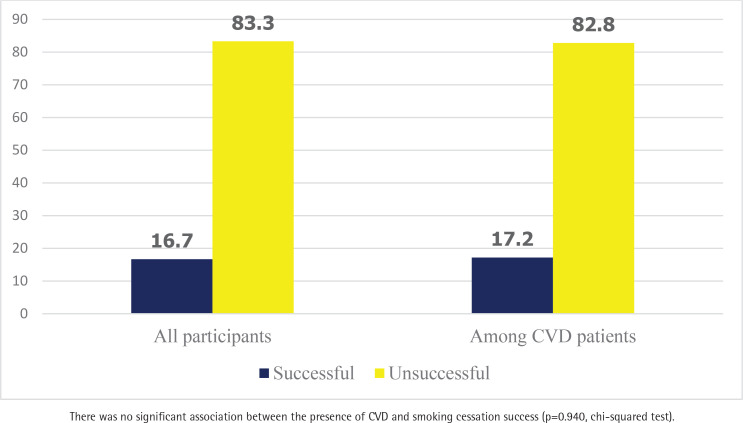
One-year smoking cessation success rates (%) among all participants (n=539) and patients with CVD (n=145), retrospective cohort study in Türkiye, 2022–2023

A total of 90 participants (16.7%) successfully quit smoking, while 449 (83.3%) were unsuccessful. Among 145 patients with CVD, 25 (17.2%) successfully quit smoking, while 60 (44.1%) completed their treatment (Figure 2).

Of the total participants, 306 (56.7%) received bupropion, 167 (30.9%) received nicotine patches, and 23 (4.2%) received combination therapy. Patients who completed treatment had a smoking cessation success rate of 33.0% (n=70), whereas those who discontinued had a success rate of 22.2% (n=20) (p<0.001). A significant association was found between bupropion use and treatment adherence; 63.2% (n=134) of bupropion users completed treatment, whereas the completion rate among nonusers was 36.8% (n=78) (p=0.015). No significant association (p>0.05) was found between other treatment types and treatment adherence (Table 1).

**Table 1:** Comparison of sociodemographic characteristics, nicotine dependence levels, comorbidities, and treatment modalities by smoking cessation success and treatment completion among adults attending a smoking cessation clinic in Türkiye, retrospective cohort study, May 2022–April 2023 (N=539)

*Variables*	*Successful*	*Unsuccessful*	*p*	*Treatment* *completed*	*Discontinued* *treatment*	*p*
**Age** (years), mean ± SD	44.92 ± 13.37	41.04 ± 12.11	0.007**^b^**	42.52 ± 12.40	41.15 ± 12.39	0.209**^b^**
**Smoking pack-years,** mr (sr)	246.04 (22143.50)	274.80 (123386.50)	0.109**^c^**	271.33 (57523.00)	269.13 (88007.00)	0.873**^c^**
**FTND score,** mean ± SD, or mr (sr)	5.46 ± 2.76	6.46 ± 2.42	0.001**^b^**	255.36 (54137.00)	279.49 (91393.00)	0.077**^c^**
	***n (%)***	***n (%)***	***p***	***n (%)***	***n (%)***	***p***
**Sex**						
Female	37 (41.1)	192 (42.8)	0.863^a^	85 (40.1)	144 (44.0)	0.415^a^
Male	53 (58.9)	257 (57.2)		127 (59.9)	183 (56.0)	
**Education level**						
High school or lower	55 (61.1)	296 (65.9)	0.451^a^	143 (67.5)	208 (63.6)	0.411^a^
University or higher	35 (38.9)	153 (34.1)		69 (32.5)	119 (36.4)	
**Income**						
Minimum wage or lower	36 (40.0)	205 (45.7)	0.385^a^	96 (45.3)	145 (44.3)	0.900^a^
Minimum wage or higher	54 (60.0)	244 (54.3)		116 (54.7)	182 (55.7)	
**Comorbidity status**						
Absent	47 (52.2)	253 (56.3)	0.547^a^	114 (53.8)	186 (56.9)	0.478^a^
Present	43 (47.8)	196 (43.7)		98 (41.0)	141 (43.1)	
**Nicotine patch**						
Yes	20 (22.2)	147 (32.7)	0.065^a^	57 (26.9)	110 (33.6)	0.098^a^
No	70 (77.8)	302 (67.3)		155 (73.1)	217 (66.4)	
**Bupropion**						
Yes	55 (61.1)	251 (55.9)	0.427^a^	134 (63.2)	172 (52.6)	0.015^a^
No	35 (38.9)	198 (44.1)		78 (36.8)	155 (47.4)	
**Nicotine gum**						
Yes	2 (2.2)	5 (1.1)	0.736^a^	2 (28.6)	5 (71.4)	0.710^c^
No	88 (97.8)	444 (98.9)		210 (99.1)	322 (98.5)	
**Combination therapy**						
Yes	4 (17.4)	19 (4.2)	0.927^a^	9 (4.2)	14 (4.3)	0.984^a^
No	86 (95.6)	430 (95.8)		203 (95.8)	313 (95.7)	
**Treatment**						
Completed	70 (33.0)	142 (31.6)	<0.001^a^			
Discontinued	20 (22.2)	307 (68.4)				
**Total**	90 (100)	449 (100)		212 (100)	327 (100)	

mr: mean rank, sr: sum of ranks.

a Chi-squared test.

b Independent t-test.

c Mann-Whitney U test. ‘Successful’ cessation refers to abstinence for at least one year. ‘Unsuccessful’ includes relapse or failure to quit. ‘Completed’ treatment indicates use of bupropion for at least two months, NRT for at least three months, or full behavioral follow-up. ‘Discontinued’ refers to early termination of any treatment.

Table 1 also presents the relationship between age, cigarette pack-years, FTND scores, treatment adherence, and smoking cessation success. No significant differences were found between age, cigarette pack-years, and FTND scores in terms of treatment adherence (p>0.05). However, age and FTND scores were significantly associated with smoking cessation success. The mean age was significantly higher in the successful group (44.92 ± 13.37 years) compared to the unsuccessful group (41.04 ± 12.11 years) (p=0.007). The mean FTND score was 5.46 ± 2.76 in the successful group and 6.46 ± 2.42 in the unsuccessful group (p=0.001). No significant difference was found between cigarette pack-years and smoking cessation success (p>0.05).

Table 2 presents the multivariable logistic regression analysis results for factors associated with smoking cessation success among all participants. Age and FTND score were entered as continuous variables without categorization, to preserve statistical power and reflect linear associations. The analysis showed that older age (AOR=1.03; 95% CI: 1.01–1.05; p=0.002), higher level of education (AOR=1.84; 95% CI: 1.03–3.26; p=0.037), lower FTND score (AOR=0.85; 95% CI: 0.77–0.94; p=0.002), and treatment adherence (AOR=0.13; 95% CI: 0.07–0.23; p<0.001) were independently associated with higher smoking cessation success. The model explained 18.6% of the variance in smoking cessation success (Nagelkerke R^2^=0.186).

**Table 2:** Multivariable logistic regression results for factors associated with smoking cessation success among all participants attending a smoking cessation clinic in Türkiye (N=539)

*Variables*	*AOR (95% CI)*	*p*
Age (years)	1.03 (1.01–1.05)	0.002
Education level	1.84 (1.03–3.26)	0.037
FTND score	0.85 (0.77–0.94)	0.002
Nicotine patch therapy	0.61 (0.34–1.10)	0.102
Treatment completion	0.13 (0.07–0.23)	0.000

AOR: adjusted odds ratio. Model adjusted for age, education level, FTND score, nicotine patch therapy, and treatment completion. Nagelkerke R²=0.186; -2 Log Likelihood=197.90. Age and FTND score were included in the model as continuous variables.

Table 3 examines the association between CVD diagnosis and various demographic and clinical factors. Patients with CVD were significantly older than those without CVD (p<0.001). Additionally, the mean cigarette pack-years was significantly higher in the CVD group compared to the non-CVD group (p<0.001). No significant difference was found between CVD and FTND scores (p=0.194). However, CVD status was significantly associated with education level (p<0.001) and income level (p<0.001). No other significant associations were observed (p>0.05).

**Table 3:** Comparison of sociodemographic and clinical characteristics between participants with and without CVD attending a smoking cessation clinic in Türkiye (N=539)

*Variables*	*CVD present*	*CVD absent*	*p*
**Age** (years), mean ± SD	50.81 ± 10.76	38.34 ± 11.23	**<0.001^b^**
**Smoking pack-years**, mr (sr)	346.89 (50299.00)	241.70 (95231.00)	**<0.001^c^**
**FTND score,** mr (sr)	284.26 (41217.00)	264.75 (104313.00)	0.194^c^
	***n (%)***	***n (%)***	
**Sex**			
Female	70 (48.3)	159 (40.4)	0.099^a^
Male	75 (51.7)	235 (59.6)	
**Education level**			
High school or lower	120 (82.8)	231 (58.6)	**<0.001^a^**
University or higher	25 (17.2)	163 (41.4)	
**Income**			
Minimum wage or lower	84 (57.9)	157 (39.8)	**<0.001^a^**
Higher than minimum wage	61 (42.1)	237 (60.2)	
**Nicotine patch**			
Yes	45 (31.0)	122 (31.0)	0.988^a^
No	100 (69.0)	272 (69.0)	
**Bupropion**			
Yes	76 (52.4)	230 (58.4)	0.215^a^
No	69 (47.6)	164 (41.6)	
**Nicotine gum**			
Yes	3 (2.1)	4 (1.0)	0.736^a^
No	142 (97.9)	390 (99.0)	
**Combination therapy**			
Yes	9 (6.2)	14 (3.5)	0.172^a^
No	136 (93.8)	380 (96.4)	
**Behavioral modification**			
Yes	12 (8.3)	24 (6.1)	0.480^a^
No	133 (91.7)	370 (93.9)	
**Treatment**			
Completed	60 (41.4)	152 (38.6)	0.555^a^
Discontinued	85 (58.6)	242 (61.4)	
**Smoking cessation outcome**			
Successful	25 (17.2)	65 (16.5)	0.940^a^
Unsuccessful	120 (82.8)	329 (83.5)	
**Total**	145 (100)	394 (100)	

mr: mean rank, sr: sum of ranks.

a Chi-squared test.

b Independent t-test.

c Mann-Whitney U test. CVD includes coronary artery disease, myocardial infarction, arrhythmia, heart failure, peripheral artery disease, valvular disease, or cardiomyopathy. ‘Smoking cessation success’ is based on self-reported abstinence for at least one year.

Multivariable logistic regression analysis examining the association between CVD, age, smoking history, education level, income level, and smoking cessation success found that only age was significantly associated with CVD status (AOR=1.09; 95% CI: 1.06–1.11; p<0.001). No significant associations were observed between CVD and smoking history (AOR=1.00; 95% CI: 0.99–1.01; p=0.371), education (AOR=0.63; 95% CI: 0.36–1.10; p=0.110), income level (AOR=0.71; 95% CI: 0.45–1.11; p=0.141), or smoking cessation success (AOR=1.33; 95% CI: 0.73– 2.46; p=0.338). The model explained 29.9% of the variance (Nagelkerke R^2^=0.299).

**Figure 3:**
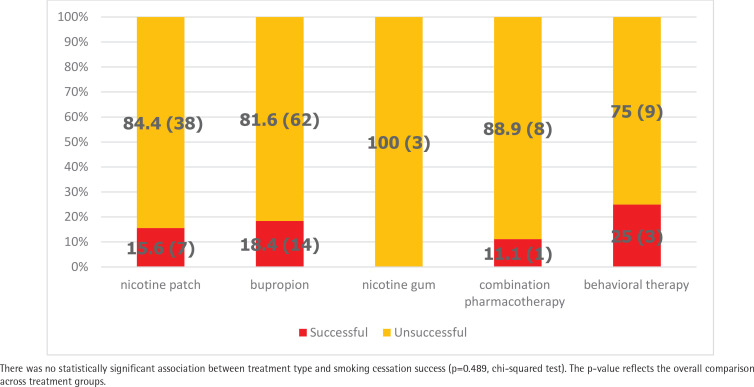
One-year smoking cessation success rates, % (n), by treatment type among patients with CVD (n=145), from a smoking cessation clinic in Türkiye, 2022–2023

**Figure 4:**
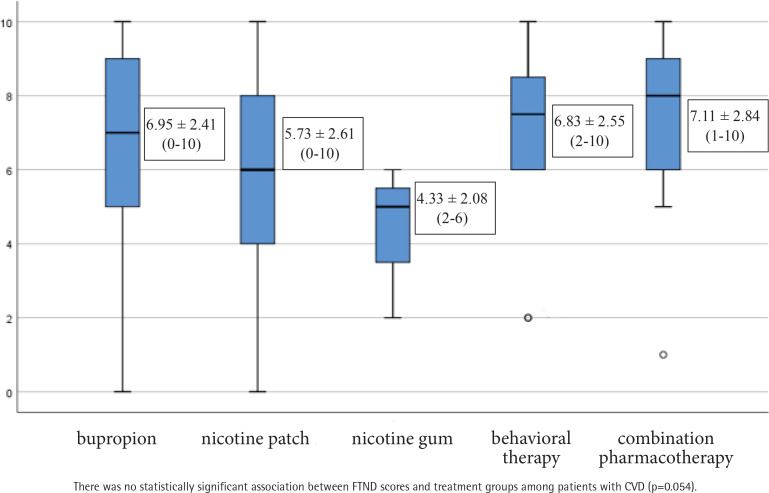
FTND scores (mean ± SD, range) by treatment type among patients with CVD (n=145), from a smoking cessation clinic in Türkiye, 2022–2023

Among CVD patients (n=145), the highest smoking cessation success rate was observed in the behavioral counseling-only group (25.0%), followed by the bupropion group (18.4%) However, no statistically significant association was found between treatment type and smoking cessation success (Figure 3). For comparisons of FTND scores across the five treatment groups, one-way ANOVA was performed and no statistically significant association was found between FTND scores and treatment groups (p=0.054). The mean ± SD values of FTND scores across treatment groups are presented in Figure 4 for patients with CVD: bupropion 6.95 ± 2.41 (range: 0–10), nicotine patch 5.73 ± 2.61 (range: 0–10), behavioral counseling 6.83 ± 2.55 (range: 2–10), combination therapy 7.11 ± 2.84 (range: 1–10), nicotine gum 4.33 ± 2.08 (range: 2–6).

Among CVD patients (n=145), smoking cessation success was not significantly associated with gender, education level, income level, FTND scores, or treatment type (p>0.05). However, according to Mann-Whitney U test, a significant association was found between smoking cessation success and age (p=0.030). The mean rank of successful patients was significantly higher than that of unsuccessful patients. Additionally, a strong association was found between smoking cessation success and treatment adherence (p<0.001). Among successful participants, 76.0% (n=19) completed treatment, whereas in the unsuccessful group, the treatment completion rate was 34.2% (n=41).

Table 4 presents the results of the multivariable logistic regression analysis examining factors associated with smoking cessation success among patients with CVD (n=145). The analysis showed that older age was independently associated with higher cessation success (AOR=1.06; 95% CI: 1.01–1.12; p=0.018), as was treatment adherence (AOR=0.15; 95% CI: 0.05–0.43; p<0.001). In contrast, education level (AOR=1.24; 95% CI: 0.34–4.56; p=0.739) and FTND score (AOR=0.90; 95% CI: 0.75–1.09; p=0.170) were not significantly associated with smoking cessation success. The model explained 24.1% of the variance in cessation outcomes (Nagelkerke R^2^=0.241).

**Table 4:** Multivariable logistic regression results for smoking cessation success among participants with CVD attending a smoking cessation clinic in Türkiye (N=145)

*Variables*	*AOR (95% CI)*	*p*
Age (years)	1.06 (1.01–1.12)	0.018
Treatment completion	0.15 (0.05–0.43)	0.000
Education level	1.24 (0.34–4.56)	0.739
FTND score	0.90 (0.75–1.09)	0.170

AOR: adjusted odds ratio. Model adjusted for age, education level, FTND score, and treatment completion. Nagelkerke R²=0.241; -2 Log Likelihood=110.56. Age and FTND score were included in the model as continuous variables.

## DISCUSSION

In this study, data were retrospectively analyzed from individuals who received smoking cessation treatment. Previous studies have reported smoking cessation rates ranging between 8% and 39%^14-18^. This wide variation is attributed to differences in sociodemographic characteristics, levels of nicotine dependence, types and continuity of interventions, and the structure of healthcare services across studies. The one-year smoking cessation rate observed in this study was 16.7%, based on self-reported outcomes, and is consistent with rates reported in the existing literature.

Numerous studies have demonstrated that smoking cessation success is influenced by multiple factors, including demographic and psychosocial characteristics, type of treatment, treatment adherence, comorbidities, and motivation^19-22^. In this study, older age, lower nicotine dependence, higher level of education, and treatment completion were significantly associated with smoking cessation success. These findings align with previous literature.

A retrospective study reported a one-year smoking cessation rate of 36.0% among individuals aged ≥65 years^17^. A prospective cohort study in Taiwan found that after three years, smoking cessation rates were 37.3% in individuals aged ≥65 years, compared to 26.5% in those aged <65 years^23^. Aging is associated with an increased burden of health issues, reduced social pressure compared to younger populations, a more stable lifestyle, lower nicotine tolerance, and greater social support from peers and family – all of which contribute to higher smoking cessation success rates^19,24,25^.

A longitudinal population-based study in Finland demonstrated that individuals with higher levels of education were more likely to quit smoking compared to those with only basic education^26^. Higher smoking cessation rates among well-educated individuals are thought to result from greater health awareness, a better understanding of smoking-related harms, and more active participation in cessation programs. Additionally, greater access to healthcare services may further contribute to higher smoking cessation success rates among this group^19,24^.

In this study, the mean FTND score was relatively high (6.26 ± 2.51), suggesting that participants had a higher level of nicotine dependence. Higher dependence levels increase withdrawal symptoms, complicate the cessation process, and lead to higher relapse rates, necessitating more intensive and individualized behavioral and pharmacological interventions^19,24,25^.

A strong association was observed between smoking cessation success and treatment adherence among all participants. However, no significant difference was found in treatment adherence rates between patients with and without CVD. In general, treatment adherence rates in smoking cessation programs range from 30% to 50%^13,27^. Enhancing adherence rates is crucial, as treatment completion is a key determinant of smoking cessation success. Adherence rates vary based on treatment type, duration, patient motivation, and potential side effects^13,28^. This study found that treatment adherence rates were significantly higher among bupropion users. Bupropion inhibits dopamine and norepinephrine re-uptake, thereby reducing nicotine withdrawal symptoms and stress-related mood changes, while also exerting an antidepressant effect. These pharmacological properties may have contributed to higher adherence rates among bupropion users^29^.

From a CVD perspective, age and treatment adherence were identified as significant key factors with smoking cessation success among patients with CVD. This finding suggests that younger patients with CVD or those with lower adherence to treatment may have a lower likelihood of successfully quitting smoking. A meta-analysis demonstrated that smoking cessation rates tend to increase with age; however, age alone is not the sole determinant. Factors such as the presence of health issues (particularly CVD), increased awareness, and social support also play a crucial role^30^.

Among younger individuals, health perception may differ, and they may not fully appreciate the longterm consequences of their disease, leading to lower adherence to smoking cessation treatments. Studies have shown that low adherence is associated with lower smoking cessation success rates^28^. Participation in comprehensive cardiac rehabilitation programs has been shown to improve smoking cessation outcomes among CVD patients^31^. Therefore, it is recommended that higher-risk patient groups receive more intensive monitoring, individualized support, and psychosocial interventions to enhance cessation success.

The EUROASPIRE IV study, conducted in Europe, evaluated smoking cessation success among patients hospitalized for cardiovascular events. In individuals with coronary artery disease, smoking cessation rates were approximately 50% after an average follow-up of 1.4 years. However, cessation rates varied significantly across countries, with reported success rates of 33% in Cyprus, 61% in Finland, and 29% in France. Smoking cessation rates were generally higher in Northern European countries, where individualized counseling, intensive follow-up, and behavioral change programs were implemented. These variations in success rates are influenced by multiple factors, including baseline smoking frequency before CVD onset, the structure and effectiveness of smoking cessation programs, tobacco control policies, cultural norms, and socioeconomic conditions^32^.

A nine-year retrospective analysis conducted in South Korea reported a smoking cessation rate of 71.7% among individuals diagnosed with cancer or CVD. This high success rate was attributed to the structure of healthcare services, greater health awareness among individuals, and the comprehensive nature of smoking cessation programs in Korea^12^.

In this study, the smoking cessation rate among CVD patients (17.2%) was lower compared to previously reported rates. One potential explanation for this discrepancy is the lack of standardized followup programs tailored specifically for individuals diagnosed with CVD in Türkiye. Studies have demonstrated that hospital-based interventions, such as the Ottawa Model for Smoking Cessation, are effective strategies for treating tobacco dependence.

Under the Ottawa Model, all patients who smoke are identified during hospital admission, receive brief counseling about the benefits of quitting, and are encouraged to engage in cessation efforts. Treatment plans are personalized based on the patient’s level of nicotine dependence and overall health status, and follow-up continues after hospital discharge. In London, patients referred to a smoking cessation specialist following hospital admission had a sixmonth cessation success rate of 35.1%. Key factors influencing success included strong motivation to quit, higher nicotine dependence, and the presence of diabetes^33^. In Canada, hospitals implementing the Ottawa Model reported smoking cessation success rates of 30–35% after one year, whereas hospitals that did not use this model had significantly lower success rates of 5–10%^34^.

In this study, CVD patients who received only psychosocial counseling had the highest one-year smoking cessation rate (25.0%) compared to other treatment groups. Similar findings have been reported in the literature^35^. The limited use of pharmacological treatments in CVD patients may have led to a greater emphasis on non-pharmacological approaches, such as psychosocial counseling and behavioral modification interventions.

### Limitations

This study has several limitations. First, smoking cessation status was based on self-reported data without biochemical verification, which may introduce recall and response bias. Second, data on the time interval between cardiovascular events and smoking cessation clinic visits were not available, limiting the evaluation of timing-related effects. Additionally, due to the observational nature of the study, causal inferences cannot be made, and residual confounding by unmeasured factors such as motivation or stress may exist. As stress and related psychological factors such as depression or anxiety can affect treatment adherence and increase the risk of relapse, the absence of mental health assessments represents an additional limitation. Selection bias is also possible, as not all eligible patients were reachable. Baseline differences in gender and nicotine dependence across treatment groups may have influenced treatment allocation and cessation outcomes. Finally, since the study was conducted in a single public smoking cessation clinic in Türkiye, the findings may have limited generalizability to other populations or countries.

### Future research

Future research should incorporate objective biochemical verification methods and evaluate the timing of smoking cessation attempts after cardiovascular events. Future studies should also consider long-term relapse beyond the first year of cessation. In addition, larger, multi-center studies with representative samples are needed to improve generalizability and to better account for potential confounding factors.

## CONCLUSIONS

This study highlights that smoking cessation success remains low among patients with cardiovascular disease (CVD), despite the well-established benefits. Older age and treatment adherence were identified as key factors associated with cessation success, while a CVD diagnosis alone was not sufficient to promote abstinence. These findings suggest that current intervention strategies may be inadequate for high-risk populations. To improve outcomes, future programs should prioritize individualized, evidencebased approaches supported by structured follow-up.

## Data Availability

The data supporting this research are available from the authors on reasonable request.
